# “From the moment I wake up I will use it…every day, very hour”: a qualitative study on the patterns of adolescents’ mobile touch screen device use from adolescent and parent perspectives

**DOI:** 10.1186/s12887-019-1399-5

**Published:** 2019-01-24

**Authors:** Siao Hui Toh, Erin K. Howie, Pieter Coenen, Leon M. Straker

**Affiliations:** 1 School of Physiotherapy and Exercise Science, Curtin University, Kent Street Bentley, Perth, WA 6845 Australia; 2 Physiotherapy Department, KK Women’s and Children’s Hospital, Singapore, Singapore; 3 Department of Health, Human Performance and Recreation, University of Arkansas, Fayetteville, Arkansas USA; 4 Department of Public and Occupational Health, Amsterdam Public Health Research Institute, VU University Medical Center, Amsterdam, the Netherlands

**Keywords:** Adolescent, Mobile touch screen device, Smartphone, Tablet computer, Qualitative research

## Abstract

****Background**:**

The use of mobile touch screen devices, e.g. smartphones and tablet computers, has become increasingly prevalent among adolescents. However, little is known about how adolescents use these devices and potential influences on their use. Hence, this qualitative study explored adolescents’ perceptions on their patterns of use and factors influencing use, and perceptions and concerns from parents.

****Methods**:**

Semi-structured interviews were conducted with adolescents (*n* = 36; 11 to 18 years) and their parents/caregivers (*n* = 28) in Singapore recruited to represent males and females across a range of ages from different socioeconomic groups. Prompts covered weekday and weekend use patterns, types of activities, perspectives on amount of use, parental control measures and concerns. Interviews were recorded and transcribed. Transcripts were coded and thematic analysis was carried out.

****Results**:**

Smartphone was the most common mobile device owned and used by many of the adolescents, while only some used a tablet. Many adolescents and their parents felt that adolescents’ MTSD use was high, frequent and ubiquitous, with frequent checking of device and multitasking during use. Reported influences of use included functional, personal and external influences. Some of the influences were irresistibility of mobile devices, lack of self-control, entertainment or relaxation value, and high use by peers, family and for schoolwork that contributed to high use, or school/parental control measures and lack of internet availability that limited use. Most adolescents were generally unconcerned about their use and perceived their usage as appropriate, while most parents expressed several concerns about their adolescents’ use and perceived their usage as excessive.

**Conclusions:**

This study has provided rich insights into the patterns and influences of contemporary mobile device use by adolescents. Mobile device use has become an integral part of adolescents’ daily routines, and was affected by several functional, personal and external influences which either facilitated or limited their use. There also seemed to be a strong inclination for adolescents to frequently check and use their mobile devices. There is an urgent need to understand the implications of these common adolescent behaviours to inform advice for wise mobile device use by adolescents.

**Electronic supplementary material:**

The online version of this article (10.1186/s12887-019-1399-5) contains supplementary material, which is available to authorized users.

## Background

In the past few years, there has been a surge in the ownership and usage of mobile touch screen devices (MTSD) among adolescents [1–3]. MTSDs refer to portable electronic devices in which users interact with a touch sensitive screen interface using their digits or a stylus pen, specifically smartphones and tablet computers [4]. Several recent large surveys have reported higher prevalence and amount of MTSD use among adolescents compared to traditional electronic devices such as television, desktop or laptop computers [2, 3, 5, 6]. This prevalence of use may be due to increased ownership, portability, ease of accessing the internet, and a variety of other functions such as social networking, gaming and shows/videos offered by MTSDs [1, 3, 7]. Furthermore, MTSDs can also offer other benefits such as delivering health information or interventions (telehealth) [8], building family time and connectedness [9], or improving ease of communication and motivation in learning [10], which may further facilitate increased use. However, this increased use has also raised concerns among parents, researchers and educators about its potential negative impact on adolescents’ mental, social and physical well-being and development, such as depression [11], adverse family relationships [12], cyberbullying [13], poor sleep quality [14, 15], sedentariness [16], musculoskeletal symptoms [17, 18] and visual symptoms [18].

To date, research on adolescents’ MTSD usage has mostly focused on the prevalence and/or duration of use, with a lack of in-depth reporting on the patterns of MTSD use, including routines, types of activities, breaks taken and nature of multitasking. Although several studies have reported that many adolescents use MTSDs frequently [2, 3, 5, 6], and commonly at night or before sleeping [19, 20], little is known about adolescents’ daily routines of using these devices, which can be affected by different types of day (e.g. weekdays versus weekend, school days versus holidays) or settings (e.g. home versus school). Another not well understood pattern of use is the nature and extent of multitasking during MTSD use. Adolescents use MTSDs for a variety of activities such as social media, messaging, gaming or video watching [1, 21], but research on how they perform and/or switch between these activities is limited. Different patterns of MTSD use, such as the type of activities or nature of multitasking, may also vary risks for various negative outcomes. For example, some studies found associated adverse outcomes of eye discomfort with video watching [12], and disrupted learning, sleep and reduced productivity with multitasking [22]. Therefore, it is important to examine in-depth the various patterns of use, to allow a better understanding of adolescents’ use of MTSDs.

Rules or restrictions from parents have been shown to help mediate adolescents’ technology use [23–25]. Most of the research, from quantitative and/or qualitative studies, has examined parental rules or restrictions for television [26, 27], internet use [23, 28, 29] or technology use in general [24, 30, 31]. Parental rules and restrictions for MTSDs may, however, be different from the traditional devices. There may be different challenges posed in view of greater portability and pervasive use of MTSDs [6], and possibly different parental concerns on adolescents’ use of MTSDs [2, 24]. Hence, it is important to examine parental rules or restrictions and concerns to understand the family context.

Moreover, there is limited qualitative research on adolescents’ MTSD use. Qualitative studies are important as they can provide rich detailed information on adolescents’ use of MTSDs, which survey research is not able to provide [32]. Therefore, this study will adopt qualitative methods to explore perspectives from adolescents and parents in Singapore on (i) the patterns of MTSD use by adolescents including routines, type of activities, breaks and multitasking and (ii) the rules, restrictions and concerns from parents/caregivers on adolescents’ MTSDs use. Whilst the focus of this study was the perspectives from adolescents on their MTSD use, perspectives from their parents were also sought in order to provide triangulation and to give greater context to data obtained from adolescents [33]. Any agreement or disagreement on perspectives between adolescent and his/her parent were also identified. Information gained from this study will allow a better understanding and deeper insights into adolescents’ MTSD use, which can help parents, educators, policy makers and researchers to develop strategies to support wise use of MTSDs by adolescents.

## Methods

### Recruitment and participants

Adolescents and their parents/caregivers (from the same families) were recruited together, via convenience sampling through personal contacts and advertisements on social media and forums in Singapore. Inclusion criteria for adolescents were: aged 11 to 18 (inclusive) years old, used any type of MTSDs, and could speak English. Recruitment was also carried out to ensure adolescents from both genders and across age groups of 11 to 12, 13 to 15 and 16 to 18 years old were represented. Participation of parents/caregivers was not a prerequisite for participation by the adolescents. Ethics approval for this study had been obtained from Curtin University Human Research Ethics Committee (RDHS-77-15). For adolescents aged 11 to 17 years, written informed parental consent and written youth assent were obtained. For adolescents aged 18 years, youth consent was obtained. Written consent was also obtained from the parents/caregivers. Recruitment continued until data saturation occurred, when no new information was being obtained across three consecutive interviews [34, 35].

### Interviews

Semi-structured interviews were conducted during June to September 2016, with both adolescents and their parents/caregivers by one of the authors (SHT) who had received training on interviews for qualitative research prior to the study. Interviews were conducted based on an interview guide with question prompts (see Additional file 1). For adolescents, questions covered types and ownership of MTSDs used, types of activities carried out, routines of use on weekdays and weekends, breaks taken, multitasking, perception of amount of usage and parental rules or restrictions. For parents/caregivers, questions covered amount of usage, rules or restrictions and concerns about their adolescent’s MTSD use. Demographics such as race, type of housing and parents’ education level (as proxy socioeconomic statuses) were also obtained. The semi-structured format was adopted as it allowed for discussion of new topics raised by participants and for any nuances to be pursued [36].

Interviews were conducted individually with each adolescent and his or her parent/caregiver in English. Attention was given to ensure that adolescents and parents were not in the same room during each other’s interviews. The majority of the interviews were carried out in participants’ homes with another location (e.g. restaurant, café or parent’s workplace) used if it was not convenient to conduct the interviews at the participant’s home. Most of the interviews lasted approximately 30 min (range: 20 to 55 min) with each adolescent and 15 min (range: 10 to 35 min) with each parent/caregiver.

### Data analysis

With permission from participants, each interview was audio-recorded and transcribed verbatim by the first author. Transcripts were coded independently by one of the authors (SHT) and reviewed by another author (EKH), using NVivo 11 software based on areas of research questions, i.e. patterns, routines, rules and restrictions and concerns of adolescents’ MTSD use. Coding for each adolescent’s transcript was done first, followed by his or her own parent/caregiver’s transcript. Adolescent and his or her parent/caregiver’s transcripts were then compared for triangulation of data [33], and separate codes on any agreement and/or disagreement between adolescents and their parent/caregiver were generated. Thematic analysis was carried out with themes generated from the codes using an inductive approach. The research questions set the broad areas for analysis, and coding and theme generation were refined throughout the analysis period [37, 38]. All themes were reviewed and discussed by all the authors, and differences in interpretation were resolved.

## Results

### Participant demographics

In total, 36 adolescents and 28 parents/caregivers (*n* = 27 parents, *n* = 1 caregiver) (from the same families) were recruited and interviewed. The mean age of adolescents was 14.2 (2.3) years. The number of parents/caregivers were less than the number of adolescents as four pairs of adolescents were siblings with another participant and their parents were interviewed once regarding both siblings. Four of the parents were not interviewed as they did not speak English. Demographics of adolescents are presented in Table 1.

**Table 1:** Adolescent participant demographics (*n* = 36)

Characteristic		Participants *n*, (%)
Age range	11 to 12 years	11 (31)
	13 to 15 years	14 (39)
	16 to 18 years	11 (31)
Schooling level	Primary school	11 (31)
	Lower secondary school	11 (31)
	Upper secondary school	6 (17)
	Post-secondary school	8 (22)
Gender	Male	16 (44)
	Female	20 (56)
Race	Chinese	27 (75)
	Malay	5 (14)
	Indian	3 (8)
	Others	1 (3)
Type of housing	2/ 3 room HDB^1^ flat	4 (11)
	4 room HDB^1^ flat	8 (22)
	5 room HDB^1^/executive HDB^1^ flat	13 (36)
	Private housing	11 (31)
Father’s highest education level	Primary	0 (0)
	Secondary	8 (22)
	Post-secondary	25 (69)
	Don’t know	3 (8)
Mother’s highest education level	Primary	2 (6)
	Secondary	10 (28)
	Post-secondary	22 (61)
	Don’t know	2 (6)

^1^HDB: Housing Development Board (public housing), with increasing size related to ascending socioeconomic status, and private housing being higher status than HDB housing

### Themes

The analysis yielded several themes which were organized by the research questions into four overall themes, with the first theme being the types of MTSDs used and owned. The second theme explored patterns of how adolescents use MTSDs and incorporate use into their daily routines. The third theme explored functional, personal and external factors that influence adolescents’ use of MTSDs. The last theme was about concerns on MTSD use. Themes and their sub-themes are presented in detail and supported with quotes from adolescents (A) and parents (P) below. Adolescents and their parents agreed on most aspects of MTSD use, except on certain influences and concerns of use where quotes from parents were also presented.

### Types of MTSDs used and owned

Smartphones were the most frequently used MTSD and were used by all adolescents. Tablet computers were used by some, and a touch screen iPod was used by one adolescent. Some used multiple MTSDs - both smartphone and tablet or more than one smartphone. Almost all adolescents owned a smartphone and used it daily, except a few younger adolescents (in primary school; 11–12 years old) who did not have their own as their parents did not allow them to have one. They usually sought permission from their parents and borrowed their smartphones to use instead.

For most adolescents, a tablet computer was used much less frequently than a smartphone, usually once or a few times per week or month. Most did not have their own tablet and used a tablet that was shared with either parents or siblings. Some adolescents did not use a tablet as much as a smartphone as they felt that a tablet had fewer functions, was harder to use for messaging and social media and also less portable than a smartphone. They usually used a tablet as a replacement when their smartphone was out of battery or malfunctioned, or to watch shows/videos or browse for information when they wanted to use a larger screen.

### Patterns of MTSD use

#### (i) High and frequent use, integrated into daily routines with frequent “checking” of device

Many of the adolescents, especially those who owned a MTSD, used MTSDs frequently throughout the whole day during weekdays and weekends, whenever possible. They used them from morning until night time, often interspersed with their daily activities and with frequent “checking” of the device. Upon waking up in the morning, they started using their smartphones to turn off the alarm, check the time and/or social media and messages, and used them again after washing up and/or during breakfast. They used them again when commuting to and from school and during school hours, usually before the start of lessons, during recess and/or lessons (if allowed by teacher). After returning home from school, they used MTSDs again, after or during lunch and/or dinner, and continued to use them during or after finishing homework. They also often reported using them again at night before bedtime, usually in their bedrooms, as these were the times that they were usually free and able to have uninterrupted use and privacy. During weekends many adolescents similarly used MTSDs throughout the whole day whenever possible, even when outdoors for school extra-curricular activities, tuition (supplementary lessons), outings with family and friends or while commuting. Examples of how they used MTSD frequently throughout the day are:

“In the morning when I eat breakfast, I use my phone, then after that [when] go[ing] [to] school [I] also use… after school [when I] come back [I] also use. Technically I every moment also use…” (A24).

“For weekends… I use [a] handphone [a] lot of time… and social media and the usual… then after that, probably would freshen up and head out to meet friends outside. Yeah throughout the whole day when I [I’m] doing activities and stuff outside, I will definitely still be using my phone.” (A23).

Throughout the day there was also frequent “checking” of smartphones for messages, updates from social media, games and/or other applications. This “checking” was reported by many adolescents and it could occur sporadically, ranging from small bursts of time for a few minutes to longer periods, for example:

“It’s more of an interval thing… open [phone] check then close, open check close… [almost] every other minute, each time less than even three minutes. Just a simple check then close.” (A23).

Many parents also agreed with their adolescents in their adolescents’ frequent use of MTSDs; they reported that their adolescents seemed to always have their MTSDs with them, using them almost the whole time whenever possible:

“She is using her mobile most of the time. So, [whenever] I see her, the mobile phone usually will not leave her… just like [it is] attached to her every time [I see her].” (P15–16).

#### (ii) Ubiquitous use

The use of MTSDs by adolescents was ubiquitous; at various locations at home, in school and in the community when outdoors, bringing them almost wherever they were and having them often within reach. At home, adolescents used MTSDs at various places, such as on the sofa or dining table, in the living room or kitchen, on the study table or in bed in the bedroom, and even in the toilet, which were similarly noted by their parents too:

“Every other minute they will not go anywhere without the phone, even from the room to the hall to the kitchen… anywhere in the house he moves, the phone moves with him.” (P28).

In school, whether in the assembly hall before school starts, the classroom during or in between lessons, or in the canteen during recess or lunch time, they also used MTSDs. When outdoors in the community, for example when commuting in public transport, in cars or when walking to and from school or other places, and when out with family and friends (e.g. shopping centres or restaurants), they also often used MTSDs. At home, many adolescents tended to use MTSDs for longer periods as they had more free time available and were not occupied with school lessons or other activities. In school and in the community, use tended to be intermittent in shorter bouts, during breaks or any free time that was available when they go about their activities; for example, before start of morning school assembly, when commuting or whilst waiting (e.g. for food to arrive when dining out or parents to finish their shopping errands).

#### (iii) Multitasking

Many adolescents reported multitasking with other tasks, e.g. homework, eating, and even during washing up or dressing, at the same time when using MTSDs. They also used MTSDs with other devices, e.g. television (often during advertisements), desktop or laptop computers. When they were multitasking, they often checked their smartphones for messages or social media updates. They also engaged in online browsing, games, video watching or listening to music, for example:

“…I will watch [TV] and at the same time I will look at my phone to check the messages. Sometimes I listen to music on my phone, then I read the subtitles on TV… I use the phone during advertisements and if like the show is boring right, halfway [through the show] I just get the main idea, then I [start to] use my phone.” (A17).

Some adolescents generally felt that multitasking MTSD use with other tasks or devices did not require “much effort” and that it was “natural” for them to do so:

“Usually when you eat out nowadays, you either watch television or YouTube on the internet. It’s actually natural, [be]cause when you eat you don’t really use your eyes. You can actually eat and look at the phone… just look once [at the phone] and then you eat, so it doesn’t really take much effort to do that.” (A35).

Use of MTSDs while doing homework was commonly reported by many adolescents. They used them for personal activities (e.g. messaging, games or music) and for schoolwork (e.g. Google translate or dictionary, searching information for projects, messaging with schoolmates to consult each other on homework).

In addition, some adolescents also reported that they often multitasked among different types of activities, by switching and alternating repeatedly among them, with short periods of use for each activity. They might switch to and from different activities due to notifications or updates received from messages, social media or other applications prompting them to switch, boredom with a particular activity or their mood (what they felt like doing at that time). For example, they might be playing games, but when they received notifications, become bored with playing games or feel like checking messages or social media, they would switch to messaging or social media and then back to games again. This process of switching was then repeated again:

“…when no new feeds or updates on the social media, then I will switch to game. Then after that, when I’m done with the game, I’ll switch back to social media, and [when] there’s incoming WhatsApp and so on, I’ll switch to that. Yeah so it’s just a constant switch around.” (A23).

Table 2 provides other example of quotes supporting the themes of high and frequent use, integrated into daily routines with frequent “checking” of device, ubiquitous use and multitasking.

**Table 2:** Findings on patterns of MTSD use

Patterns of MTSD use	
(i) High and frequent use, integrated into daily routines with frequent “checking” of device	“From the moment I wake up I will use it to check my messages, and then also [to] check the time, like before I go school, I check how much time [is] left and all. Then I will use my phone in school to check when the next period is, cause I saved my timetable in my phone. Then I use iPhone to communicate with my friends, if I need to find them I will then text them. Then maybe like on the way to school, I will listen to music using my phone, even during my Chinese orchestra practices [school extra-curricular activity]… so basically there’s like a lot of functions for me to use. It’s like every day, every hour [I] definitely will be using it.” (A17) “Saturday and Sunday I got [have] tuition… I wake up in the morning then after that I use, eat breakfast and use my phone also. Then [when] go tuition, I’ll also use the phone on the bus … After I come back from tuition, I just start doing my homework. Sometimes [when] I’m bored during the middle [of homework], I will also use [phone].” (A24) “If it’s a weekend, then normally I [will] have time to use [phone]. I’ll probably use my phone before I sleep at maybe like 10 o’clock.” (A25) “Yeah, just before I sleep I use [phone] in my room because I want to check my WhatsApp. Then after that I sleep.” (A26) “After school, I go back [home] and open Wi-Fi. Check [if] got any messages, sure [to have] got [messages] after so long. Surely got twitter messages coming. If no messages, I’ll just watch some videos.” (A11) “After waking up, I’ll check the time again. Then check social media, watch some YouTube videos, [and] read a bit more.” (A25) “When they come into the house…the first step is they [will] go and on the Wi-Fi. So that means that they want to use it [phone] all the time. And then they will see messages from friends or whatsoever. So this is their lives, part of their lives.” (P18)
(ii) Ubiquitous use	“Use it [phone] anywhere, just bring my cup and use my phone at the same time” (A7) “…once I reach school, the next time I will be using it [phone] will be [during] lunch...then I will check messages or any others…should be around 5 to 10 min only because the rest of the time I would be talking to my friends and eating.” (A12) “Wherever she walk her phone must be with her, even [when] go[ing] [to] toilet, [she] also must bring [phone].” (P2)
(iii) Multitasking	“[When] I brush my teeth, the video [on phone] is playing... when I put on the buttons on my school uniform, I will also watch videos [on phone]. Then sometimes when I do homework I also use [phone].” (A30) “Sometimes I listen to song [on phone] when doing work [homework]. Sometimes if I am not listening to song, I [will] just watch video, put the video [phone] over here…and do work [homework].” (A11) “...we use Google document to do our project on the computer, but then we [also] use WhatsApp to talk. I don’t know why, WhatsApp is the easiest… For Google document, we just type out whatever we need, but then we [also] use WhatsApp to communicate...” (A17) “…use my phone and do my homework at the same time…to check for some word meaning that I don’t understand, like go [to] Google and search for the definition of the word.” (A11)

### Reported influences of MTSD use

Several influences of MTSD use were reported by adolescents and parents, which could be categorized into functional, personal and external influences:

#### (i) Functional influences

##### Device performance and internet availability

Poor device performance and lack of internet access or mobile data can limit the amount of time spent and activities available to use on MTSDs. A few of the adolescents mentioned that they seldom used their older models of tablet or smartphones as they were slow in loading applications and websites, or they might malfunction halfway when using. Moreover, some adolescents had no internet/Wi-Fi access at home, outdoors, in school or public places and/or limited or no mobile data plan on their MTSDs. They were thus limited in their use of social media, messaging, online games or browsing on their MTSDs, and were only able to make phone calls, play games or watch videos that they had already downloaded on their MTSDs. Adolescents who lacked a data plan on their MTSDs often tried to access the internet at places that had Wi-Fi access, such as in school, public areas or areas in the house where Wi-Fi connection was available or stronger.

Portability of device could also affect the types of MTSD used at various locations. Both smartphones and tablet computers were often used at home by the adolescents; but in school or in the community, a smartphone was often brought and used outdoors instead, as it was more portable than a tablet. A tablet was usually brought to school only if it was needed for school lessons.

##### Multiple functions and activities

Many adolescents reported using MTSDs for a wide variety of functions and activities, ranging from personal and schoolwork to daily life functions. Personal activities commonly included social activities such as messaging using WhatsApp, social media on Facebook, Instagram, Snapchat or Twitter, and making phone calls. They also used MTSDs to browse the internet on areas of interest e.g. entertainment idols or strategies for computer games, read online fiction or shop online. Other common activities included watching shows/videos ranging from a few minutes to a few hours on YouTube, video streaming websites or downloaded videos, playing games and listening to music. They also used them for school related matters, e.g. during school lessons, searching information for projects and communication with classmates and teachers to consult on homework. MTSDs were also frequently used for daily functions, e.g. checking time, setting a wake-up alarm, checking directions on map or transport arrival time and taking photographs.

##### Entertainment or relaxation

Some of the adolescents used MTSD during any available leisure or free time, either as a form of entertainment or relaxation to reduce boredom or to kill time. Some of the instances mentioned were free time before or after lessons, during recess, after finishing homework, before sleeping or whilst waiting e.g. for meals when dining out or for transport to arrive. They used MTSDs to keep themselves entertained or occupied, as they had “nothing else to do”, or were generally “bored” with the tasks that they were doing, such as homework:

“Because when I’m eating, there’s nothing [else] to do. So I will be quite bored, so I will just like watch videos or read… if you just sit down and eat [also] very boring, so I [will] also use my phone.” (A24).

“I do my work, and then after a while, I use my phone. Then I will do my work again, then I use my phone and I do my work again… When I don’t know how to do the question or [when] I’m like bored, I will quickly use it, [be]cause [I] lose interest in the work. Then what else can you do? You [I] just use the phone.” (A21).

Homework was one of the common tasks where adolescents reported using MTSDs intermittently. MTSD use was often perceived as a form of break or relaxation from homework. A few even mentioned that they were in fact able to concentrate better on their homework after or while using MTSDs. Interestingly, a few adolescents also mentioned that they eventually ended up getting bored with using MTSDs after some time, although they were kept occupied and entertained by them initially, for example:

“You know on that weekend where you got absolutely nothing to do, it’s boring. Because you are not going to school, you’re not doing anything, you’re not going out… you’re just staying at home and doing absolutely nothing but using the phone. It’s very boring. Sooner or later you will become bored [with the phone] and it is obvious.” (A13).

#### (ii) Personal influences

##### Irresistibility of MTSDs

To many of the adolescents, MTSDs seemed irresistible such that they often felt an inclination to use MTSDs, even more so when MTSDs were within their sight and reach. This appeared consistently and is a strong theme that emerged from the data. There was also a sense of attachment and dependency on MTSDs, especially smartphones, to the extent that they were always with them or within their reach, and “one day without phone [I] cannot survive” (A36). For example:

“...it’s quite tempting because you are always using it, then you suddenly don’t use it for several hours, you will tend to want to pick it up and look. For me I’ll just look through [phone], then after [when] I’m done with the things, I’ll put [phone] down.” (A8).

In addition, it appeared that many adolescents also had a strong inclination to check their smartphones. They reported frequent checking of their smartphones throughout the day, and often receiving beeping from notifications or updates from messages, social media or other applications, which prompted them to check and use their smartphones. There was also regular influx of notifications of new messages, especially those from group chats. These notifications were at times distracting but the adolescents were still inclined to check them as they wanted to keep updated with their peers and of events happening around them, and “not want to be left out” (A28). Otherwise, they might feel uncomfortable or not able to concentrate on the tasks that they were doing:

“I don’t know, I feel uncomfortable if I don’t look at my phone, there will be lots of messages going out to me. I can’t just ignore it. I feel uncomfortable [if] I don’t look at it before I fall asleep, so I reply a bit on whatever that [messages] is happening, then I go to sleep.” (A28).

Adolescents’ inclination to use MTSDs was also evident when some of them attempted to plead or ask for permission from their parents, despite parental rules or restrictions on duration or periods of use:

“Sometimes [when] I finish halfway I [will] want to continue, but the time limit is running out, so I have to [switch] off. Sometimes I will ask my parents [I want to] play a little bit more please. Sometimes they will say yes, but sometimes no.” (A18).

##### Lack of self-control

Many adolescents appeared to have a lack of self-control over their use whereby they found it hard to resist or stop using MTSDs. They expressed that they tended to get carried away when using MTSDs and lose track of time. For example, when they are watching shows, they are often inclined to continue watching another episode to the extent that their homework or sleeping time was delayed:

“When you watch shows, these shows actually have series and so after you watch one episode, you will just want to watch the rest. So that part is quite hard to overcome, so that’s why I become addictive.” (A8).

“…bad to the extent that I would not sleep on time…I only sleep at 3 or 4 am and then after that the next day, I have to wake up at 7 o’clock [for school].” (A10).

Some adolescents reported that they attempted to implement self-control measures and exercise self-discipline on their MTSD use, as they felt that they were overusing them. Self-control measures mentioned included putting MTSDs out of reach, setting a timer or alarm to use for limited durations, using applications with reminders to discourage unlocking devices, ignoring messages or notifications that were unimportant, and avoiding certain activities (e.g. shows/videos that they tend to get carried away with). However, some of these adolescents reported difficulties with adhering to the measures, as they sometimes also became carried away with what they were doing on their MTSDs and ended up overusing them:

“I put it [phone] outside if not I will keep on using. Cause once you start using, it can go on for like 3 hours just sitting there and using…then after another 2 hours I will come out and use my phone again. Sometimes it depends, sometimes I get carried away with my phone, end up using phone for like half an hour then never go back and do work [homework].” (A17).

#### (iii) External influences

##### Schedule differences affect the amount of time available to use MTSDs

The amount of time that adolescents spent on MTSDs was variable from day to day, mainly due to their different schedules of activities, which affected the amount of free time available to use MTSDs. Adolescents who were more occupied on certain weekends and weekdays with more homework, studying especially during exam periods, school extra-curricular activities, tuition, sports or outings with family or friends reported that they did not have as much time available to use MTSDs, and hence did not use them as much as other days when they had more free time available. For example:

“On Saturday and Sunday, I don’t actually use my phone that much because in the morning I have activities. I go Aikido [martial art], then when I come back I usually have some stuff to do like assignments… so I don’t have much spare time in between... I usually use it for about one and a half [hour] after dinner, because I’m completely occupied before dinner.” (A35).

During weekends and school holidays, most adolescents reported using MTSDs for longer durations than during school weekdays. Some adolescents even used MTSDs for longer periods before bedtime which delayed their sleep, as they did not have to wake up early the next day for school.

The amount of free time available also affected the types of activities carried out on MTSDs. For example,some adolescents mentioned that they were only able to check messages or notifications from social media when they were free for short durations (e.g. when preparing to go to school in the morning or waiting for transport) but watched shows/videos when they had longer durations available (e.g. after finishing homework at home).

##### High use among peers and family members, and for school related matters

Many of the adolescents frequently reported high MTSD use among their peers and family members (parents, siblings and/or relatives), and the need to use MTSDs to communicate with their friends, family, schoolmates and even teachers (e.g. to discuss homework or school projects or obtain updates about school events or lessons). A few parents have also pointed out that they spent considerable amount of time on MTSDs themselves, for example:

“We parents get carried away as well. So when we get carried away, they [adolescents] also see and will say: okay, now is the time I [adolescent] can also use phone right?” (P26).

Some adolescents also reported that they and their peers frequently used smartphones and even messaged each other when they were together. MTSDs were also used to play multiplayer games, watch shows/videos, share and take photos together or others, such that it becomes a social activity. Some parents also agreed with their adolescents about the prevalent use of MTSDs among peers and for school related matters, which made it difficult for them to control or limit their adolescents’ use:

“So, we cannot totally stop it [phone use], we also have to take care of her sensitive…her feelings you see...so we kind of got to balance that. She will be faced with all the peer pressure, and this is the time when she makes friends. She is learning how to socialize, so we cannot cut that off totally.” (P18).

##### Control measures by the school and parents/caregivers

All the adolescents reported that their schools have rules on MTSD use in school, which limited the amount of their use during school hours. Adolescents were generally compliant with these rules and were allowed to bring MTSDs to school, but not allowed to use them during lessons unless allowed by teachers. A few adolescents mentioned that their schools even disallowed MTSD use during recess or allowed its use only at certain common areas in the school. There were penalties such as confiscation of MTSDs if adolescents disobeyed the rules. For the adolescents in post-secondary schools, the rules seemed to be generally less strict; they were able to use MTSDs at any area in the school, and at any other time except during lessons unless allowed by teachers.

Some parents/caregivers reported implementing rules or restrictions on their adolescents’ MTSD use and were strict in ensuring that they observe them, especially for the younger adolescents (in primary and lower secondary school). Other parents were more relaxed about the rules or did not enforce any, especially for the older adolescents, as they required more autonomy and greater use of MTSDs for communication with peers and schoolwork.

The majority of parental measures implemented were restrictions on duration (e.g. setting time limit, disallowing use during certain periods such as meals, before finishing homework or exam period), access to MTSDs (e.g. parents keeping away MTSDs at night, not providing adolescents with their own MTSD), types of activities or applications (e.g. permission required to download games or post on social media), or amount of internet data plan on MTSDs. Some adolescents reported being compliant with the measures at times which helped to prevent them from using MTSDs excessively.

##### Non-compliance with parental control measures

Although some adolescents were compliant with parental control measures, the compliance was not consistent and there were often instances of non-compliance reported by parents and adolescents. Some adolescents reported attempts to hide from their parents’ supervision when using MTSDs, e.g. using MTSDs in a locked bedroom or secretly switching on home Wi-Fi access. Hence, parents were sometimes unsure if their adolescents were compliant with the measures.

In addition, the younger adolescents (in primary school) who did not have their own MTSDs, as their parents did not allow them to have one, often tried to find ways to access their parents’ MTSDs without asking or obtaining permission. For example, they took their parents’ MTSD to use when their parents were occupied or tried to hack the password that it was locked with:

“…sometimes they manage to hack it [password]… they will see the pattern that we punch so they can guess. Sometimes they manage to get the first number and last number, then they start guessing [the other numbers].” (P36).

Many parents expressed difficulties and frustrations in implementing rules or restrictions on their adolescents’ MTSD use, and in ensuring compliance and appropriate use of MTSDs even after relaxing or removing the rules. Some parents mentioned that they often had to “tell” their adolescents to stop using MTSDs; to the adolescents, this was often perceived as “nagging” by their parents which they had become accustomed to hearing. If adolescents still did not comply even after repeated “nagging” or reminders, some parents eventually relented on their rules or restrictions. For example:

“I will tell them that [to stop using] but eventually if they don’t listen to me, then I think it is no point in doing the nagging…not worth it, no point. I have said too many times already… and he [adolescent] don’t practice [listen], it is meaningless to worry, so don’t go to the extend.” (P19).

“Sometimes my mother will keep on nagging. Never stop nagging… sometimes I will listen and go study, sometimes I just continue using.” (A27).

It was difficult for parents to regularly monitor their adolescents’ MTSD use and ensure compliance with the rules or restrictions, especially when they were away at work or when adolescents were in school or out by themselves. A few parents articulated that it was important for adolescents to be aware of and exercise self-discipline on their usage, as they were not able to control their use all the time, for instance:

“I mean we can only remind this much…control this much right? They have to realise for themselves, whether through the hard way or the easier way of listening to advice. The hard way is [for them to] see their [exam] results, know already then they will really wake up and try to put things right.” (P25).

Table 3 provides further examples of participant quotes on influences of MTSD use.

**Table 3:** Findings on influences of MTSD use

Influences of MTSD use	
(i) Functional influences	
Device performance and internet availability	“When I go home, there is no internet so I don’t really use it [tablet]. So if I’m bored I will play like the offline games, but I don’t play for very long, maybe half an hour.” (A6) “Because my school doesn’t have a wireless Wi-Fi, so I have to use my own data to go to social networks. I like to go to Instagram but Instagram really takes up a lot of data so I can’t go much.” (A8)
Multiple functions and activities	“I can use my phone to do a lot of things. I mainly use my phone to watch YouTube and use WhatsApp...it’s convenient cause it’s nice for watching. When I need to check something else I can Google and WhatsApp… and [go] back and forth between them.” (A35)
Entertainment or relaxation	“… when parents ordering the food [in restaurant], I will play [phone] for a while [be]cause I’m bored. When the food come already, I won’t [use], I will keep [it].” (A27) “When I do homework after one hour I will watch one short video, then I go back to work…[be]cause sometimes when I do my work [homework], I will get very bored you know.” (A20) “I will go do my work [homework]. Then after a while, I will come out and use my phone again… then I go and do my work. When cannot take it already, then I use my phone again… I will use it to SMS, then afterwards I will use it to look at Twitter, Instagram.” (A17)
(ii) Personal influences	
Irresistibility of MTSDs	“My pants [have] almost all no pocket[s]. So I have to hold my phone in my hand…hold[ing] in your hand has [created] temptation to keep using [the] phone.” (A1) “Sometimes people will spam me [with] messages, so I have no choice but to reply them until I feel tired…” (A11) “I’ll just check my phone when messages come. Check [phone] already, then won’t be distracted as much, phone makes you know what is happening around [in] the group chat.” (A1) “I usually use WhatsApp or that kind of group chat...because I don’t want to be like left out in a way. I want to know what’s going on, [so] I’ll just look.” (A28) “When I’m doing homework, the phone can be distracting [be]cause usually in group chats, they [friends] will usually message us, and you will always see the messages popping out.” (A8) “He always finds [a] reason to use [phone]. He will ask for permission in between his homework [to use]… he got a lot of reasons.” (P29)
Lack of self-control	“I’ll take and just play every day, busy playing until almost forget homework.” (A36) “Sometimes I overstretch what I should be doing, for example I accidentally spent 1 or 2 h on the phone when I should be doing my homework… basically is when I get into the chain of watching YouTube videos, or when reading this [online] book [be]cause the book is very long.” (A25) “There’s this particular blog right, I watch most of their videos. I find it very nice, so I watch it over and over again or find more of these videos [be]cause they seem interesting…then I cannot stop, so I keep on using.” (A34)
(iii) External influences	
Schedule differences affect the amount of time available to use MTSDs	“During weekend, if I need to wake up early the next day, I will just sleep at 11. But if don’t have to, we can stay up until like maybe 12 or 1 then we sleep. So, I use my phone until then… also depends on if I’ve a lot of homework [to do] or not.” (A30)
High use among peers and family members, and for school related matters	“Sometimes like when my parent’s friends come to play with us, their kids right, we all play Minecraft together [on tablet or phone] for quite long, sometimes from 12 am to 3 am.” (A15) “We have like WhatsApp groups for the class to update us about the next day school or homework and stuff. Sometimes the teacher will post the homework online for us to do, and sometimes we also have to do to hand it up online, so I will [have to] use the phone.” (A21)
Control measures by the school and parents/caregivers	“School only allow [us] to use phone during recess in the canteen…outside canteen, we cannot use.” (A11) “Can only use [phone] before school, during recess and after school. If you are found using [phone] during lessons, it will just get confiscated.” (A4) “There are a few restrictions [from parents] like you can’t download anything, and if we like break the time limit, then we might have to…I don’t know…maybe face consequences like get scolded or something.” (A14)
Non-compliance with parental control measures	“During night time when I sleep, he hides inside the room. He locks the door, I also don’t know if he uses phone or not.” (P4) “She will purposely hide the fact that she is using her phone. Now I think she can’t be bothered because now when I’m at home, she will just use it in front of me. I tried to tell her not to use your phone for like Facebook or Instagram, they are just to trap people but she never listens.” (P8) “Our phone has got password. So sometimes if I happen to use it and then I didn’t lock it, and [when] I put it down and he sees it, he will grab it and run away [with it] and hope you don’t notice.” (P5) “I told them, that’s why I told them. But you know right, when you don’t see them, even when you see them, if they are doing that [using tablet or phone] so often, I myself am sick of telling them [not to use].” (P19) “When I saw that they are sticking to the discipline, then I stop the policing [of their phone use]. But after that I noticed they are not really obeying to our rule already, they slowly slipped back to their old habit.” (P34) “The father has set up some rules for them, but I [am] hardly at home, so I seldom see whether they do [follow] it or not… he said that there’s a schedule for them [to use phone], when to when, what time to what time, but I really don’t know whether they practice it or not.” (P15–16) “My mum is constantly working so she is mostly not at home. So, I just keep using [phone]. No one to control me.” (A4)

### Concerns on MTSD use

#### (i) Concerns from adolescents and parents

The adolescents generally did not raise many concerns on their MTSD use; only a few mentioned that their eyesight might have been affected from it. Some adolescents did not usually keep track of the amount of time spent using and tended to use MTSDs continuously for long periods without taking breaks. Breaks were usually perceived by the adolescents as the time when they stopped using MTSDs, rather than a conscious effort to take a break. Hence, some adolescents were not conscious of taking breaks and usually stopped using only when they need to carry out other tasks or activities:

“No, I don’t consciously take a break to rest my eyes. I just keep on using until maybe I’m like tired or something then I’ll off the phone, so I don’t usually tell myself “ok stop [using]”. For the iPad, I don’t think I took breaks…I just keep on using it until I had to stop because of other reasons like my friends calling me to go outside.” (A8).

On the other hand, almost all of the parents expressed concerns regarding their adolescents’ MTSD use, and were worried about possible negative impact on their adolescents’ mental health and behaviours (e.g. energy levels, ability to focus, time management or tendency to violence), physical development (e.g. eyesight, postures, bodily discomfort, physical activity level), social development (e.g. social skills, family bonding) and exposure to inappropriate content online or bad peer influence (e.g. cyberbullying).

#### (ii) Perspectives on amount of use

Most adolescents perceived their amount of MTSD use as appropriate and did not see the need to change or limit their use. They felt that their usage was appropriate as they implemented self-control measures on their MTSD use or did not spend as much time on MTSDs as compared to their friends. On the other hand, a few adolescents perceived their usage as excessive but did not take any active measures to control their usage, as they felt that it was difficult to change their habits of use:

“…it’s too much, but I also cannot help it, you cannot help me…I see [phone] again I forget about [that] I use too much also.” (A29).

“Too much, of course too much, but it’s a bit hard to you know, to stop it [be]cause it becomes an addiction.” (A15).

There were disagreements between adolescents and their parents on the amount of use; most parents perceived their adolescents’ usage as excessive which was often in contrast with their adolescents’ perception. A few parents reported that the contrasting perceptions had even caused unhappiness and discord between them and their adolescents, for example:

“…there is a disagreement that we think she use the phone too much, she thinks she use it just nice. So, this cannot come to an understanding, that’s why always have quarrel between us. Yeah so, we have certain rules, then she not happy, we also not happy.” (P3).

## Discussion

In this qualitative study, the rich descriptions by both adolescents and parents provided insights into MTSD use by adolescents including perceived high, frequent and ubiquitous MTSD use, multitasking as a common activity, factors that influence MTSD use, and the perceived appropriateness of their use by adolescents and parents.

### High, frequent and ubiquitous MTSD use

Both adolescents and parents perceived adolescents’ MTSD use to be high, frequent and ubiquitous, especially that of smartphones, which were consistent with recent survey studies [1, 3, 6]. Many adolescents used MTSDs ubiquitously at various locations, and frequently throughout the day upon waking up in the morning, in school and at home, even when outdoors or while commuting. Night time or before bedtime use in their bedrooms were also a standard routine of use for many adolescents, which is consistent with other studies showing prevalent technology use at night or before sleeping [2, 3, 20]. These patterns of use, integrated into daily routines, may explain findings from previous survey studies of how adolescents were able to have high total technology use, of up to nine hours a day on average [3], and smartphone and tablet being the devices with the highest amount of use compared to other devices [18]. This integration of use into their everyday life has indeed provided benefits such as convenience, communication with peers and family or access to information for homework [39]. However, if such usage becomes excessive or problematic, it can be detrimental to their academic performance, social relationships [40], mental [11] and physical health [18]. Their MTSD use is expected to increase even further with time [41]. Future studies therefore need to examine what the right balance of MTSD use is for adolescents.

In addition, this study has also highlighted a common pattern of MTSD use among adolescents - multitasking during MTSD use, which is consistent with survey studies indicating increased prevalence of multitasking with multiple devices in adolescents [1, 42]. The most common way of multitasking reported was using smartphone, for homework or personal activities (e.g. messaging or social media) while doing homework, which was also commonly observed among adolescents in a nationwide survey study in USA [3]. This high use of multitasking might be due to convenience, useful tools, communication with peers for homework, relief from fatigue or boredom from homework, or while waiting for an activity (e.g. shows/videos to load). Other studies have also found that adolescents perceived multitasking as enjoyable [43], effortless and an integral part of life [44]. However, negative outcomes such as impaired learning [45], reduced academic performance [42], poorer social and psychological well-being [46] have been associated with multitasking. Much about the nature and impact of multitasking during MTSD use in adolescents still remains unknown; further research on the appropriate extent of multitasking MTSD use with homework, other tasks and devices is therefore needed.

### Influences of MTSD use

Data from this study has also suggested several influences that facilitate adolescents’ high, frequent and ubiquitous MTSD use, which could be categorized into: functional (portability, internet availability, multiple functions and activities, entertainment or relaxation), external (peers/family/school use) and personal (irresistibility and lack of self-control) influences. Portability of MTSDs has enabled adolescents to use MTSDs ubiquitously; almost everywhere and anywhere was possible, even in various areas of the house including the toilet as reported by adolescents and parents. Availability of internet, coupled with the multiple functions and activities offered especially when online, including use for entertainment or relaxation to occupy themselves, further encouraged adolescents to use MTSDs.

The findings also suggest that the high use of MTSDs among peers and family members has an influence on adolescents’ use of MTSDs. Many adolescents felt the need to frequently use these devices, usually for messaging or social media, in order to communicate and feel involved with their peers. MTSDs were also used as a social activity for example, to play games or watch shows/videos when together with their peers or family. This is supported by other studies which have found that feelings of belonging, peer pressure [47], and frequent use of technology by parents were associated with increased technology use by children [23, 48].

Irresistibility of MTSD use to the adolescents and their lack of self-control were also reported influences that may account for their high and frequent use. Findings in this study strongly suggest that some adolescents were very attached to and unable to resist using their MTSDs, especially smartphone. Some of them reported frequently checking their MTSDs, unable to exercise self-control to stop using or resist using MTSDs when they were supposed to do homework or other tasks, or comply with parental rules or restrictions. Previous quantitative studies similarly showed high frequency of checking and inclination to use; with adolescents checking their phones an average of 150 times per day [49], and a large proportion of adolescents checking at least once an hour and feeling “addicted” to their MTSDs [6]. Although this study did not examine addiction nor had any criteria to identify those who were addicted to MTSD use, some of the adolescents’ descriptions on their inclination and lack of self-control to resist using MTSDs were suggestive of addictive behaviour. One of the reasons for this behaviour was the notifications or updates received on MTSDs, which many adolescents felt the need to respond to immediately [6]. It is also important to consider the types of activities that the adolescents engage with on MTSDs that draw them into continued use. Social networking and media use, playing games [50, 51] and watching shows [50] were related to increased risk for dependence or addiction to smartphones. Moreover, the use of social media and other activities on phones have also been found to release feel-good neurochemicals such as dopamine [52, 53]. Each use of MTSD may therefore promote reward seeking behavior that could lead to compulsive device use, and distraction or irritability when adolescents are separated from their phones. Adolescents’ brains were more responsive to reward than adults, which may thus make them more vulnerable to addiction than adults [54]. With a lack of self-control, and excessive attachment and inclination to use MTSDs, they may be even more susceptible to excessive MTSD use. Addiction to MTSD use should be avoided as it can result in detrimental effects such as stress, depression or reduced physical activity [55, 56]. There appears to be a need to address this irresistibility of MTSDs reported by the adolescents, and develop strategies to help adolescents use MTSDs appropriately without putting them at risk of addiction.

### Implications for research and practice

#### Control measures on MTSD use

Findings from this study have raised some important implications for further research and practice related to developing strategies for school, parents and adolescents to support wise MTSD use by adolescents. In this study, control measures by schools appeared to help reduce the amount of time and activities that adolescents can carry out on their MTSDs. Whilst this control from schools may limit overall MTSD use, it makes integration of MTSDs into education difficult, and is not preparing adolescents to be adults in charge of their MTSD use. Further research is required on how schools and adolescents can prevent misuse of MTSDs while maximizing benefits for education.

Adolescents whose parents had implemented control measures, such as restrictions on ownership, access to types of activities and/or duration of use had limitations in their use of MTSDs. Adolescents who did not have their own MTSD (younger adolescents in primary school), appeared to have less usage than those who owned a MTSD. This is supported by a survey study which showed an association between ownership of devices and higher sedentary time involving technology use [57]. Parental restriction of access to internet and mobile data also limited time spent on social media or content online by adolescents. This is consistent with other studies which have indicated mediation of children’s technology use by parental rules or restrictions [25, 48]. Both school and parents/caregivers thus play an important role in regulating adolescents’ use of MTSDs.

This study also highlights the fact that many parents/caregivers had difficulty implementing control measures and ensuring compliance from adolescents. One of the most common difficulties reported was being away at work most of the time and not being able to monitor adolescent’s MTSD use, possibly due to Singapore having a high percentage of dual income working parents [58]. Portability and the variety of activities and social media platforms available on MTSDs made it even harder for parents to monitor or limit their use. Contrasting perceptions on the amount of use between parents and their adolescents found in this study might also contribute to non-compliance from adolescents. Most parents in this study seemed to adopt measures that were restrictive on the usage rather than attempting to communicate or seek mutual agreement with their adolescents. Earlier research has shown that authoritative and restrictive ways of parental mediation of technology use do not contribute well to compliance [29, 59]. Moreover, parents should also moderate their own MTSD use, as their use can have an impact on adolescents’ technology use [23, 48]. Future research should thus examine effective strategies for parental mediation and seeking mutual understanding between parents and adolescents on MTSD use.

#### Lack of self-control

This study has also pointed out a general lack of awareness, concerns and self-control of MTSD use from the adolescents, which could have contributed to high and frequent MTSD use, and non-compliance with parental rules or restrictions. Studies have indicated that control measures from school and parents/caregivers can only limit adolescents’ technology use to a certain extent [60]; strategies aimed at effectively reducing screen time should target self-motivation [61]. It is thus important to increase adolescents’ own awareness of MTSD use, empower them to take charge and self-manage their usage. As seen from our data, incoming notifications, messages or updates received were often a source of irresistibility to use MTSDs. Strategies to help them self-manage their use should therefore target the incoming notifications or messages, such as silencing them or setting time limits to attend to them. Other self-management strategies may involve avoiding or setting time limits on activities that tempt continued use e.g. social media or watching shows/videos, putting MTSDs away and out of reach, use of applications to lock devices or deter its use, or setting appropriate goals e.g. using only after finishing homework. It is important for adolescents to ultimately be able to appropriately self-manage their MTSD use and bring forth good habits of use into adulthood.

Adolescents also reported that it was natural for them to turn to using MTSDs when they were free as they had “nothing to do” which were consistent with previous findings [62]. Engaging in alternative activities that are non-screen based, such as sports, hobbies or extra school activities might help to reduce the amount of MTSD use. However, it might be challenging to do so as adolescents may find screen technology use more appealing than other activities. Future studies should also examine strategies that gain adolescents’ interest and encourage engagement in more non-screen activities.

#### Measurement of MTSD use

Our findings raise some important issues for consideration in the measurement of MTSD use. Data from this study showed that patterns of MTSD use are different from traditional electronic devices. MTSD use may be continuous for long periods or of sporadic short periods, and breaks are often perceived as the time when they stopped using MTSDs instead of a conscious effort to take breaks. Patterns of MTSD exposure may also be different on different types of days e.g. weekdays, weekends, holidays and exam period. In order to capture accurately patterns of MTSD use in self-reports or other techniques, there is a need to consider patterns over time, the frequent sporadic short periods of use as well as multitasking among multiple devices.

### Strengths and limitations

Strengths of this study include a fairly large number of participants, and the semi-structured individual interview format which allowed free expression and exploration of arising issues. Moreover, perspectives from parents were sought which helped to triangulate data obtained from the adolescents and to indicate important discrepancies between the perceptions of the adolescent and the parent. One of the limitations of this study was that it reflected use at the time of data collection, but with technology hardware and software constantly changing, future use patterns might be different. In addition, during interviews with a few adolescents, their parents were sometimes within hearing distance in surrounding areas (e.g. common areas in the house), hence they might have felt unable to express their views freely. However, given that data saturation was achieved, along with the diversity and richness of information obtained, we are confident that perspectives provided by the adolescents truly reflects their opinions.

## Conclusions

This qualitative study has provided useful insights and rich information on patterns and influences of adolescents’ MTSD use, as well as implications for future research. Many adolescents and their parents felt that adolescents’ MTSD use were high, frequent and ubiquitous. Use of MTSDs was integrated into adolescents’ daily routines, often involving multitasking with other tasks or devices. There also seemed to be a strong inclination for adolescents to frequently check and use their MTSDs. Several influences of MTSD use were reported which either facilitated or limited adolescents’ MTSD use. There is a need to establish good habits of MTSD use during adolescence, so as to maximise their benefits for learning and education while minimizing any potential adverse impact on their health and development. Future research should focus on developing guidelines for wise use, and effective strategies for self-control and parental mediation of adolescents’ MTSD use.

## Additional file

Additional file 1: Interview guide and question prompts. (DOCX 15 kb)

## Abbreviation

MTSD: mobile touch screen device
