# Supplementary material for: “From the moment I wake up I will use it…every day, very hour”: a qualitative study on the patterns of adolescents’ mobile touch screen device use from adolescent and parent perspectives
Source: BMC Pediatr. 2019 Jan 24;19:30. doi: 10.1186/s12887-019-1399-5 (PMC6346550; doi:10.1186/s12887-019-1399-5)
Supplement: Supplementary file 1 — Interview guide and question prompts. (DOCX 15 kb) [file 12887_2019_1399_MOESM1_ESM.docx]

**Interview guide and question prompts**

**Adolescent**

- What types of mobile touch screen devices (MTSD) do you use? any device with touch screen such as tablet computers, smartphones?
- Are there any other types of device that you use, e.g. television, desktop and laptop computers or game consoles?
- Can you describe to me your routine of your use of tablet computer/ smartphones/ other MTSD on a typical *weekday?*
- How often and for how long?
- What activities do you use it for?
- Where do you use it?
- Can you describe to me your routine of your use of tablet computer/ smartphones/ other MTSD on a typical *weekend day?*
- How often and for how long?
- What activities do you use it for?
- Where do you use it?
- Do you usually use your tablet computer/ smartphones/ other MTSD for a long continuous time or do you take breaks in between? Why and why not? If so, how often do you take the breaks?
- What do you do during the breaks? *(sedentary or non-sedentary type of activities?)*
- Do you ever multitask - use your tablet computer/ smartphone/ other MTSD while you are doing something else, such as homework or computer? If yes, what type of devices?
- How do you multitask? What are you mainly doing when you multitask? And why do you multitask?
- Do you think your duration and frequency of use of tablet computer/ smartphones/ other MTSD is appropriate, too little or too much? Why do you think so?

**Parent/caregiver**

- What types of mobile touch screen devices does your child use? Does your child use any other forms of technology such as television, desktop or laptop computers, game consoles?
- How often does your child use his or her tablet computer/ smartphone/ other MTSD?
- What type of activities does your child carry out on his or her tablet computer/ smartphone/ other MTSD?
- What do you think of your child’s amount of MTSD use? Is it appropriate, too little or too much?
- Do you have any concerns regarding your child’s use of MTSD?
- Are there any agreements/rules in your household regarding the use of MTSD by your child, for example:
- Any on ownership or accessibility to MTSD
- Any on duration of use
- Any on use during certain days of the week or periods of the day
